# Thermo-Optical Characterization of Therminol55 Based MXene–Al_2_O_3_ Hybridized Nanofluid and New Correlations for Thermal Properties

**DOI:** 10.3390/nano12111862

**Published:** 2022-05-30

**Authors:** Likhan Das, Khairul Habib, Kashif Irshad, Rahman Saidur, Salem Algarni, Talal Alqahtani

**Affiliations:** 1 Department of Mechanical Engineering, Universiti Teknologi PETRONAS, Bandar Seri Iskandar 32610, Malaysia; likhan.das11@gmail.com; 2 Interdisciplinary Research Center for Renewable Energy and Power Systems (IRC-REPS), King Fahd University of Petroleum & Minerals, Dhahran 31261, Saudi Arabia; 3 K.A. CARE Energy Research & Innovation Center at Dhahran, Dhahran 31261, Saudi Arabia; 4 Research Centre for Nanomaterials and Energy Technology (RCNMET), School of Engineering and Technology, Sunway University, Petaling Jaya 47500, Malaysia; saidur@sunway.edu.my; 5 Department of Mechanical Engineering, King Khalid University, Abha 61413, Saudi Arabia; saalgarni@kku.edu.sa (S.A.); talqahtani@kku.edu.sa (T.A.)

**Keywords:** MXene, nanocomposite, nanofluid, thermal conductivity

## Abstract

The current research focuses on formulating a new class of Therminol55-based nanofluids that incorporates an MXene/Al_2_O_3_ nanocomposite as the new class of dispersant at three different concentrations of 0.05, 0.10, and 0.20 wt%. The optical and thermophysical properties of the formulated nanofluid are assessed experimentally. Zeta potential and FTIR analyses are employed to evaluate the composite particles' surface charge and chemical stability, respectively. Thermal conductivity is observed to increase with nanoparticle loading and maximally augmented by 61.8% for 0.20 wt%, whereas dynamic viscosity increased with adding nanoparticles but remarkably dropped with increasing temperature. In addition, the prepared TH55/MXene + Al_2_O_3_ samples are thermally stable up to 200 °C according to TGA analyses. Moreover, the proposed correlations for the thermal conductivity and viscosity showed good agreement with the experimental data. The study’s findings suggest that the formulated nanofluid could be a viable contender to be used as a heat transfer fluid in the thermal sector.

## 1. Introduction

During the last several decades, thermal engineering has advanced to the point where the heat transfer performance of heat exchangers has steadily improved. Many industry activities, including power generation, chemical processes, heating and cooling processes, transportation, microelectronics, and other micro-sized applications, rely on heat transfer fluids such as water, mineral oils, and ethylene glycol (EG) [1]. The employment of passive and active technologies, for instance, extended surface (fins and microchannels), surface vibration, fluid suction and injection, and electrical/magnetic fields, has come to a halt in order to improve heat transfer. As a result, innovative technologies with the potential to improve the thermophysical properties of conventional fluids have been a popular topic of study [2]. The suspension of various types of small solid particles, such as metallic, non-metallic, and polymeric particles, in conventional fluids to generate colloidal suspensions is an innovative technique for increasing the heat transfer performance of ordinary fluids [3]. Suspended particles on the order of µm, on the other hand, can pose serious issues in flow channels, increasing pressure loss and forcing particles to settle out of suspension quickly. In recent years, with the advancement of modern nanotechnology, several nano-sized particles were successfully synthesized with outstanding thermophysical and rheological properties [4]. Particles of nanometer dimensions dispersed in base liquids are called nanofluids. This term was first introduced by Choi in 1995 at the Argonne National Laboratory [5]. The ultra-fine nanoparticles are normally smaller than 100 nm and have remarkably higher thermal conductivity than base liquids. Despite the fact that nanofluids have exciting promise and can be utilized to replace ordinary fluids in sophisticated thermal applications, they are still in their infancy. This is due to the fact that various difficult difficulties must yet be addressed before they can be implemented in a real system. One of the most serious issues is the lack of the dispersion stability of the nanofluid. The nanofluids tend to agglomerate and therefore lose their stability after a certain period of time. To date, almost every metal, metal-oxide, nanotube, carbon-based nanoparticle, and 2D material has been examined in conjunction with various base fluids, however, the vast majority of them were demonstrated to be unstable [6,7,8]. The development of a nanocomposite-based nanofluid has recently piqued researchers’ attention in resolving this problem [9]. Nanocomposite-based nanofluids have various advantages over metallic and non-metallic nanoparticles, including increased surface area, hydrophobicity/hydrophilicity, designability, and so on. In an experiment, nano-diamond (ND) +Ni-based nanocomposite was synthesized and dispersed in a binary solution of water:EG at various ratios [10]. The thermophysical properties of the formulated nanofluids were investigated showing that the maximum enhancement in thermal conductivity (TC) was 21% compared to water, which was obtained by adding nanocomposite with a concentration of ~3 wt%. However, the dispersion stability of the nanofluid was not reported in their study. In another study, an MWCNT (Multi-wall Carbon Nanotubes)–Fe_3_O_4_ nanocomposite was incorporated to directly analyze the hydrothermal performances of a flow tube fitted with an insert turbulator [11]. They found a maximum improvement of 32.72% in the Nusselt number and a 115% penalty in the friction factor. The study, however, did not include any measurements to explain the thermophysical characteristics and dispersion stability of the nanofluids. The viscosity of nanofluids containing Ag supported on silica was reportedly lower than that of nanofluids including unsupported silica as demonstrated in the experimental work [12]. This could be attributed to silver nanoparticles becoming immobilized on the silica, preventing the formation of the three-dimensional network that is typically developed by silica. However, the major drawback of the study was that the nanofluid was found to be stable for approximately one hour, which literally indicates its unfeasibility for application in a real system. In another recent study, Ag nanoparticles were fixed onto 2D graphene nanosheets to form Ag–GNs nanocomposites, which were then distributed in EG to form a stable nanofluid [13]. When compared to the base fluid, thermal conductivity increased by roughly 32.3%. Several other studies have recently been published that demonstrate that using nanocomposites rather than traditional metallic or non-metallic nanoparticles has a higher potential in terms of long-term stability [14], thermophysical [15], chemical, optical [16], and rheological capabilities [17]. MXene is a recently invented 2D-structured material family that is similar to graphene in appearance [18]. Researchers have taken notice of MXene because it possesses exceptional electrical and thermophysical capabilities, among other properties. It is also highly flexible, with the ability to fine-tune the properties of both the material and its nanocomposites [19]. It was found to be a promising nanomaterial to promote the thermophysical properties of the heat transfer fluid in some recent demonstrations [20,21].

Since nanofluids have been widely utilized to evaluate the performance of a variety of thermal systems, both experimentally and statistically, it is required to establish correlations in order to estimate their thermophysical properties. Nanofluid-assisted systems are widely used for the performance investigation, optimization, and feasibility assessment in a numerical manner utilizing different CDF (Computational Fluid Dynamics) interfaces such as ANSYS, COMSOL, TRYNSYS, and ALTAIR for a fraction of the cost of experimental study. For better accuracy of the simulations, the properties of the nanofluids are keyed in terms of correlations of different variables (temperature, concentrations, etc.). However, some generalized models, for instance, the Maxwell model (Maxwell, 1890), and Hamilton Model (Hamilton and Crosser, 1962) cannot precisely predict the properties of the nanofluids. As a result, it is necessary to establish empirical correlations for nanofluids that take into account the type of nanoparticles, their size and shape, concentrations, and their interactions with the base fluid.

Prior research has concentrated mostly on the development of water-based nanofluids, which are not suitable for medium-temperature applications due to their thermal instability. In several investigations, oil-based nanofluids with various stabilizing agents (surfactants) were developed to increase dispersion stability. It is worth remembering that most surfactants cannot sustain higher temperatures and lose their efficacy once they reach the working temperature. As a result, formulating surfactant-free nanofluids remains a challenge, particularly for applications in the higher temperature range. In this present study, we formulated a well-stabilized Therminol55-based nanofluid using an MXene + Al_2_O_3_ nanocomposite using a two-step method. The optical, thermophysical, and rheological characteristics of the nanofluids were assessed at three different concentrations of 0.05, 0.10, and 0.20 wt%. A significant augmentation in thermal conductivity was observed, together with a small penalty in viscosity. Empirical correlations were developed to predict the thermal conductivity and viscosity showing good agreement with the experimental data. This present nanofluid could be a potential candidate for numerous thermal systems owing to its superior thermo-optical properties.

## 2. Materials and Methods

### 2.1. MXene + Al_2_O_3_ Nanocomposite Preparation

MXene nanoparticles when directly dispersed into TH55 cause the resultant nanofluids to lose their stability soon after the preparation. Therefore, MXene NPs are decorated with the Al_2_O_3_ nanoparticles which provide the resultant nanocomposites with some functionalized group that aids in better maintaining dispersion stability. In the study, three-dimensional MAX phase Al_2_O_3_ nanoparticles were purchased from Sigma Aldrich (Merck KGaA, Darmstadt, Germany) and US Nanomaterials Ltd. (Huston, TX, USA), respectively. The purity and product specifications according to the supplier catalog are provided in Table 1. On the other hand, TH55, which was used as the base fluid, was purchased from the International Rasayan Company (Punjabi Bagh, Delhi, India). The density of the TH55 was 868 gm/cm^3^ with a boiling point 351 °C as described in product catalog. The synthesis of 2D (wo Dimensional) Ti_3_C_2_ (MXene) from 3D (Three Dimensional) Ti_3_AlC_2_ (MAX) phase is in accordance with the methods described in our previous paper [22]. In the first step of MXene+ Al_2_O_3_ synthesis, 50 mg of as-synthesized MXene was dispersed into the deionized (25 mL) water and stirred for 1 h in a hot plate magnetic stirrer at 60 °C and 1000 rpm. After that, the sample was sonicated for 2 h at a frequency of 40 kHz and 500 W power. The same procedure was executed to obtain a 25 mL Al_2_O_3_(50 mg) dispersion. Later, the MXene and Al_2_O_3_ dispersions were mixed and sonicated for another 30 min with gradually adding NaOH until the pH of the dispersion reached a value of 10. Centrifuged at 4000 rpm, the resulting precipitate was carefully washed with deionized water and dried for 24 h at 70 °C to obtain the MXene + Al_2_O_3_ nanocomposite. Finally, the nanocomposite was heat-treated for 3 h at 350 °C. The MXene: Al_2_O_3_ ratio used in this investigation was 1:1 since it appeared to have the highest dispersion stability of all the ratios we investigated, for instance, 1:2, 2:1 ratio. As a result, at all concentrations, a 1:1 MXene: Al_2_O_3_ ratio was considered while forming nanocomposite.

### 2.2. Characterization Methods

**Figure 1:**
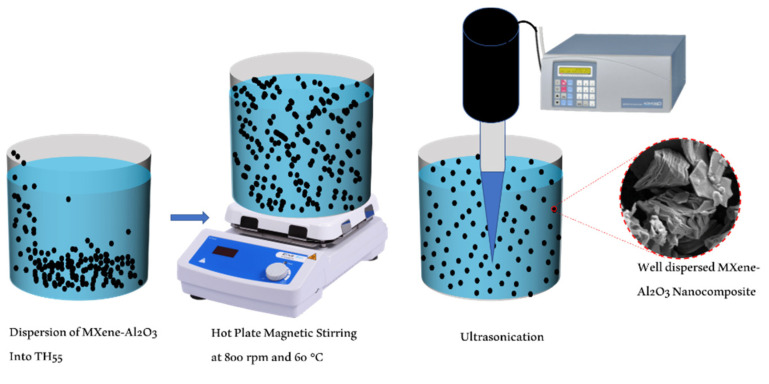
Two-step preparation of TH55/MXene + Al_2_O_3_ nanofluid.

TH55/MXene + Al_2_O_3_ hybrid nanofluid was formulated by employing the two-step preparation method, as illustrated in Figure 1. The synthesized MXene + Al_2_O_3_ nanocomposite was dispersed into Therminol-55 at loadings of 0.05, 0.10, and 0.2 wt%. Afterward, the nanofluid samples were physically stabilized utilizing a magnetic stirrer and ultra-sonicator. Stirring was performed for 45 min at 800 rpm and 60 °C subsequently, ultrasonication was conducted at 500 W, 40 kHz for 60 min per sample. Ultrasonication is an eminent technique to produce homogenous suspension breaking the agglomeration formed by the dispersed particles. No chemical additives (i.e., surfactants) were used during the formulation process of the nanofluids.

### 2.3. TH55/MXene + Al_2_O_3_ Nanofluid Preparation

The surface morphology and chemical composition of the synthesized MXene and MXene + Al_2_O_3_ nanocomposite were characterized by SEM (Scanning Electron Microscope) (Tescan, Model: Vega3, Brno, Czech Republic) and EDX (Energy Dispersive X-ray), respectively. FT-IR (Perkin Elmer^®^, Model: Spectrum Two^™^, Waltham, MA, USA) was employed to characterize the nanofluid’s existing functional groups. With an optical resolution of 0.5 cm^−1^, this device offers highly accurate data in the wavelength range 350–8300 cm^−1^. Optical absorbance and transmittance of the samples were evaluated utilizing a high-performance UV–Vis spectrophotometer (Perkin Elmer^®^, Model: Lambda 750, Waltham, MA, USA). The zeta potential of the nanofluids was measured to obtain information on their dispersion stability using a particle analyzer (Anton Paar, LITESIZER 500, Graz, Austria) with a precision of ±10%. Thermal conductivity (*k*) is determined using the transient hot wire (THW) method by thermal property analyzing equipment (Tempos from METER group, Pullman, WA, USA). A 6 cm KS-3 sensor (diameter 1.3 mm, length 60 mm) was inserted into the sample which was immersed in a water bath to assess the *k* at various temperatures. This sensor is particularly suitable for the *k* measurement in the range of 0.02–2 W/m·K providing a higher accuracy (>90%). The dynamic viscosity and rheological behavior of fluids were determined using a rheometer (Anton Paar, Model: MCR 92, Graz, Austria). Prior to performing the measurement, the instrument was calibrated with water and verified that it maintained an accurate temperature within the temperature range investigated. The thermal degradation profile of each sample of nanofluids is determined using thermogravimetric analysis via a TGA (Thermogravimetric Analysis) analyzer (Perkin Elmer^®^, Model: TGA 4000, Waltham, MA, USA). The device includes an aluminum crucible and provides precise temperature readings up to 1000 °C. Each sample requires approximately 15 mg of sample to develop a TGA profile at a heating rate of 10 °C/min. To confirm the accuracy of collecting data, all optical and thermal measurements were repeated numerous times.

## 3. Results and Discussion

### 3.1. SEM and EDX Analysis

**Figure 2:**
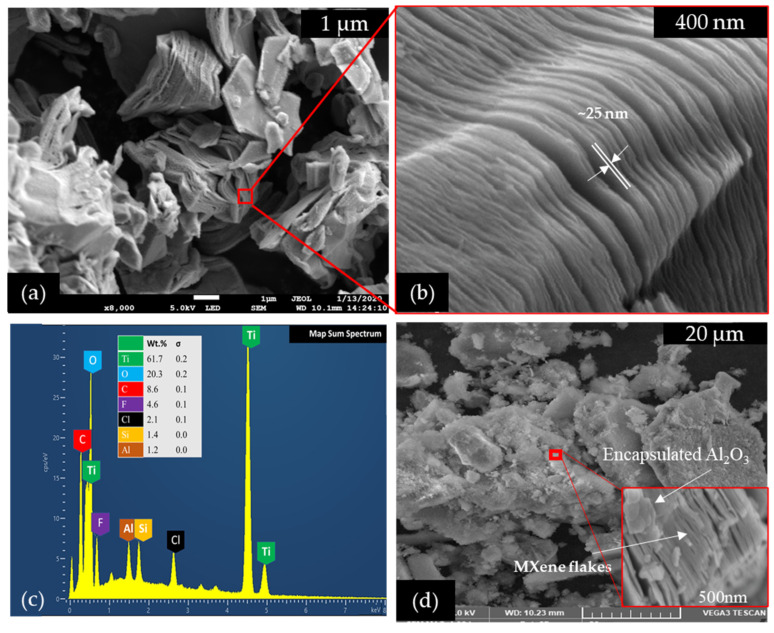
SEM of: (**a**) synthesized MXene at low magnification; (**b**) layered structure of MXene nanosheets at high magnification; (**c**) EDX analysis of the MXene; (**d**) SEM of MXene + Al_2_O_3_ nanocomposite.

The SEM images of synthesized MXene are shown in Figure 2. As evidenced by the SEM scan, the “Al” layer was successfully eliminated from the Ti_3_AlC_2_ using the HF etching method during a protracted etching process of approximately 24 h. As illustrated in Figure 2a,b, each layer of MXene is staked to the adjacent layer on both sides by the extremely strong M–X bond [23]. The average size of the MXene stacked layers varied between 1–3 µm with a lateral thickness of each layer of roughly 25 nm, as evidenced by Figure 2a,b. The EDX mapping confirms that the synthesized MXene consists of 61.7% Ti, 8.6% C, 20.3% O, 4.6% F, and a small amount of Cl, Si, and Al. The details of color mapping of the EDX analysis can be found in our published work [24]. The Al_2_O_3_ nanoparticles utilized in this study are mostly spherical in shape and have an average particle size of 50 nm with an alpha content of 80% and a gamma content of 20%, respectively. They were purchased from Us Research Nanomaterial, Inc. MXene–TiO_2_ nanocomposites with an even deposition of Al_2_O_3_ nanoparticles on the surface of MXene nanoparticles are depicted in Figure 2d. The encapsulated Al_2_O_3_ on the Mxene nanolayer substrate may have a greater specific surface area than the Mxene nanolayer itself, as demonstrated in an early study with an MXene–Ti_3_C_2_ nanocomposite [25]. It is worth noting that the higher specific surface area has advantages in improving heat transfer capability and solar light-capturing capability [26,27].

### 3.2. Zeta Potential, UV–Vis and FTIR Analysis

**Figure 3:**
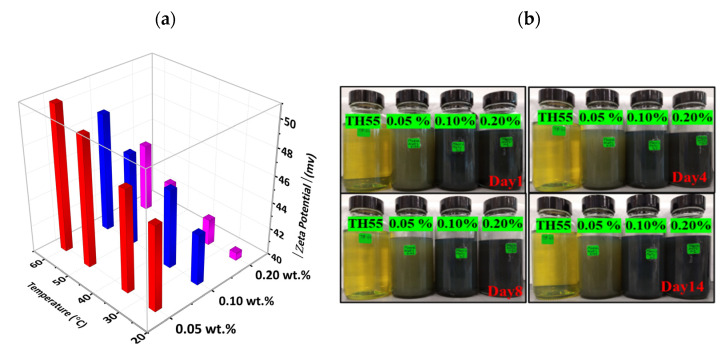
(**a**) Zeta potential of TH55/MXene + Al_2_O_3_ at different temperatures and concentrations, (**b**) digital photographs of formulated nanofluids from day 1 to day 14.

Suspensions with a Zeta potential (ζ) value of more than ±30 mV are considered electrostatically stable, and above ±60 mV indicates very good stability [28,29]. Nanofluid undergoes temperature changes in a variety of applications. With regard to nanofluids employed as coolants, they draw heat from a hot liquid, which causes them to heat up. Nevertheless, the heated nanofluids must be cooled before being circulated to the thermal system. When incorporated as coolants in heat exchangers, nanofluids undergo numerous cycles of heating and cooling. Therefore, it is essential to assess the temperature effect on the dispersion stability of nanofluid. In this study, for all concentrations, the measured |ζ| value was higher than 40 mV indicating that the formulated nanofluid is well stable at each concentration (Figure 3a). The negative value of the ζ indicates that the interfacial surface of the nanocomposites is negatively charged. However, for a stabilized dispersion, the total negative charge must be neutralized with the same number of positively charged ions in the stern and diffuse layer, which is theoretically unattainable from the nonpolar TH55 without adding stabilizing agents. In this regard, the stability of the formed nanofluid might be attributed to the formation of new stabilizing species from strong ultrasonication treatments via cavitation-induced micro-explosions. The oxidations of TH55 during the ultrasonic treatment help to form some long-chain oxygenated species such as fatty acids and other carboxylic acids, which work as the stabilizing agents of the charged particles in a liquid medium. The stabilized nanoparticles in the non-polar liquid media can be observed in previous studies without adding any surfactants [30,31,32]. However, nonfluid demonstrates comparatively reduced stability at the higher concentration (0.2 wt%), possibly due to the agglomeration of the nanomaterials in the oil medium at higher concentrations. The impact of temperature on the ζ is also noteworthy. As demonstrated in Figure 3a, the ζ increase with the rise in the studied range of temperature for each concentration. The higher ζ values at elevated temperatures are attributed to the increased Brownian motion of the solid nanoparticle. The increased Brownian motion results in an increased dynamic (vibrational/rotational/translational) motion of the nanoparticles, leading to a more stabilized form in the base fluid. The visual inspection of nanofluid stability further supports the higher values of the ζ. As shown in Figure 3b, nanofluids exhibited no substantial agglomeration or sedimentation from day 1 to day 14, indicating that the nanofluids are very stable.

**Figure 4:**
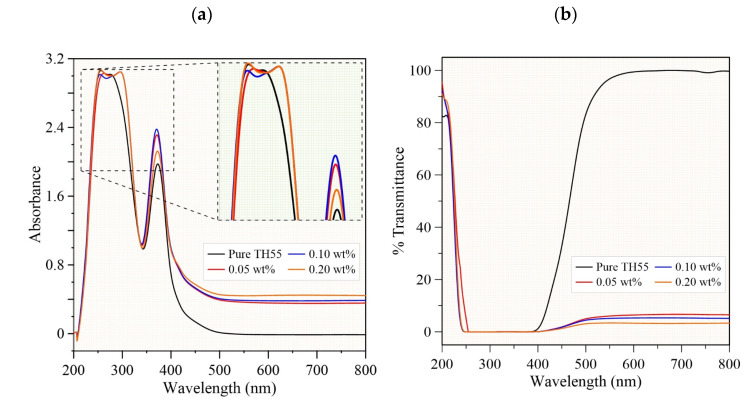

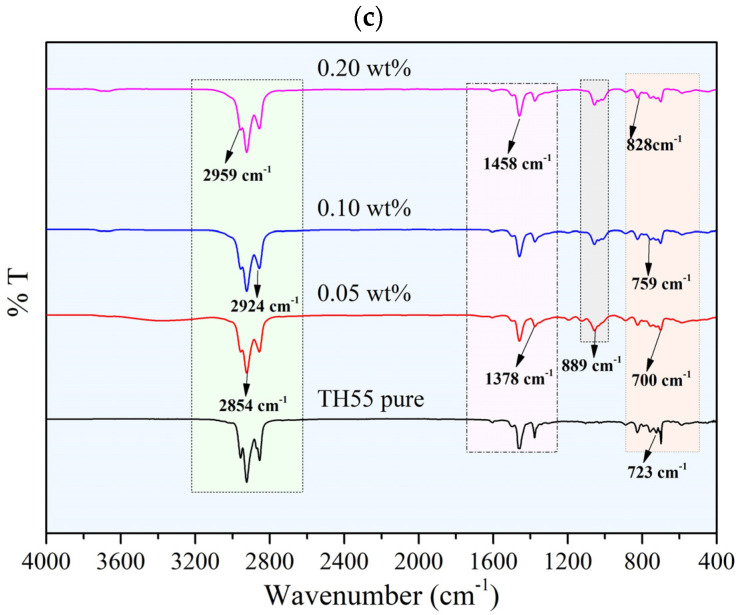
UV-Vis (**a**) Absorbance and (**b**) Transmittance spectra of hybrid TH-55/MXene + Al_2_O_3_ nanofluids at different particle loadings, (**c**) FT-IR spectra of hybrid TH-55/MXene + Al_2_O_3_ nanofluids at different particle loadings.

The UV–Vis analysis was performed with a resolution of 0.4 nm for wavelengths spanning from 200 to 800 nm. Owning to the dispersed MXene + Al_2_O_3_ nanocomposite, the UV–Vis spectra of the nanofluids showed a rise in absorbance and a decrease in transmittance of TH55 at visible wavelengths (400–800 nm), as evidenced by the trendline in Figure 4a,b. The higher capability of two-dimensional MXene flakes to absorb light resulted in an increase in optical absorption as well as a decrease in transmittance [33]. In addition, from day 1 to day 14, the optical absorbance was monitored to detect if there was any change in the absorbance peaks as a result of particle agglomeration/sedimentation, which might be attributable to the particles’ orientation changing over time. As can be seen in Figure S1a–c (Supplementary Materials), all of the peaks are nearly identical in all of the samples, with the exception of a little alteration after day 7 for the concentration of 0.2 wt%, which could be a result of moderate agglomeration at high concentrations. Nonetheless, similar peaks in all cases show that the scattered nanoparticles were not subjected to severe sedimentation or agglomeration, which would otherwise induce significant changes in the optical behavior of the fluids.

For further clarification on the chemical stability of the nanofluids, FT-IR analyses are conducted (see Figure 4c). FT-IR chemical analysis is observed from 400–4000 cm^−1^ frequency at a constant scanning speed to examine functional groups and chemical interactions of the suspensions. Prominent peaks are identified and it can be observed that the inclusion of nanomaterials resulted in a new vibration peak in nanofluid samples at 1054 cm^−1^ while other peaks remain indistinguishable as in pure TH-55. The peak appearing at 1054 cm^−1^ can be ascribed to a strong C-OH stretching bond, which might be attributed to the newly formed carboxylic acid via long-chain oxidation under an MXene/Al_2_O_3_ environment during the ultrasonication [34]. Apart from this, no chemical inconsistencies (i.e., reactions) or no shifts in existing peaks are detected among the suspensions. Additionally, escalation in transmittance offers superior photo-thermal effectiveness for the nanofluids [21]. Thus, it can be stated that the formulated nanofluids are well stable in terms of chemical characteristics.

### 3.3. Thermal Stability

**Figure 5:**
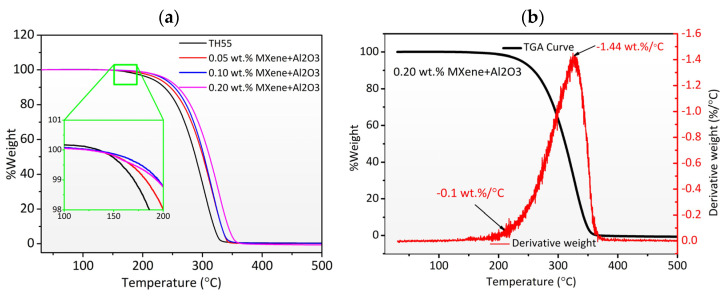
(**a**) Thermal decomposition as a function of temperature of pure Therminol55 and nanofluids at different MXene + Al_2_O_3_ concentrations, (**b**) Derivative of TGA curve of 0.20 wt% MXene + Al_2_O_3_ nanofluid.

The thermal degradation of the pure TH55 and formulated nanofluids at different concentrations are presented in Figure 5a. As demonstrated from the TGA curves, the inclusion of MXene + Al_2_O_3_ nanocomposites did not influence the decomposition behavior of the nanofluids. Therminol55 and TH55/MXene + Al_2_O_3_ nanofluids both exhibited single-step disintegration behavior, with no TGA curve discrepancies attributable to an exothermic or endothermic reaction. The TGA profiles of the pure TH-55 and nanofluid samples show high-temperature stability up to 200 °C, confirming their applicability as a heat transfer fluid in various thermal energy systems from low to medium temperatures. Furthermore, the results showed that the initial mass loss occurred at nearly 200 °C and until 250 °C partial evaporation of the TH55 can be observed. When the temperature reached 350 °C (boiling point of the TH55) the TH55 content is fully evaporated, and the residuals are the remaining nanocomposite contents. However, the addition of nanocomposite had only a slight influence on the base fluid due to low concentrations. Moreover, chemical reactions are unlikely due to an almost identical trend in the profiles. The plot of the derivative TGA curve indicates that the maximal rate of mass loss was approximately 1.44 wt%/°C and occurred in the higher temperature regime between 300 and 350 °C, whereas, at the initial stage of mass loss it was as low as −0.10%/°C. The findings confirm the nanofluids’ potential application in medium-temperature thermal implementation. Analogous investigations show a similar TGA trend for the nanofluid-based working fluid in thermal applications [21,35].

### 3.4. Thermal Conductivity

**Figure 6:**
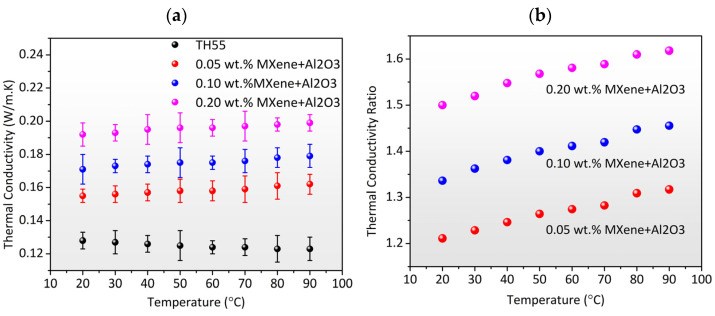
(**a**) Experimental thermal conductivity vs. temperature of Therminol55 and TH/MXene + Al_2_O_3_ nanofluid at different concentrations; (**b**) Thermal conductivity ratio vs. temperature of the nanofluids at different concentrations of MXene + Al_2_O_3_.

Thermal conductivity (TC) is an important property of the working fluid that leads to the improvement of thermal systems. The experimental TC of the as-formulated TH55/MXene + Al_2_O_3_ hybrid nanofluid was assessed in the temperature range from 20 to 90 °C at 0.05, 0.10, and 0.20 wt% concentrations. The obtained values are plotted as a function of temperature and compared with that of pure Therminol55, as depicted in Figure 6a. As evidenced from the plotting, the TC of the nanofluids experienced a substantial enhancement with the inclusion of solid nanocomposites. For the case of pure Therminol55, the TC is in the range of 0.128–0.123 W/m·K in the studied temperature range. The addition of MXene + Al_2_O_3_ by as little as 0.05 wt% led to the enhancement of the TC to 0.155–0.162 W/m·K. The impact of the concentrations of nanocomposite is also significant as can be seen from the trendlines. The higher concentrations of MXene + Al_2_O_3_ resulted in higher TC values, for instance, the TC of the nanofluids is in the range of 0.171–0.179; 0.192–0.199 for 0.10 and 0.20 wt%, respectively. This is attributed to the superior TC conductivity of the solid nanocomposites as compared to Therminol55. The interactions between the base fluid and the dispersed nanocomposite also played a vital role in the resultant TC of the nanofluid. With the advantage of the higher specific surface area of MXene, the present study demonstrates that the MXene + Al_2_O_3_ nanocomposites exhibit somewhat higher TC conductivity than other TH55/metal-oxide-based nanofluids [36,37]. The effect of temperature on the TC of the TH55/MXene + Al_2_O_3_ nanofluid is less influential than nanocomposite concentrations. However, the behavior of the nanofluids is opposite to the pure Therminol55. The pure Therminol55 showed a decrease in the TC as the temperature rises in line with previous studies, whereas, for the case of TH55/MXene + Al_2_O_3,_ an opposite trend is reported for all concentrations. This is a widespread phenomenon of nanofluids and can be explained by the fact that the increased Brownian motion at higher temperatures results in a greater capacity for heat transmission among the nanoparticles [38]. Nonetheless, the added vibrational and rotational motions of nanoparticles may contribute significantly to heat transfer between the nanoparticles and the surrounding fluid. Figure 6b illustrates the thermal conductivity ratio (TCR), *k_nf_/k_bf_* at varying temperatures. It is obvious that the TCR is increased with adding MXene + Al_2_O_3_ nanocomposite. However, the maximum enhancement was observed at concentrations of 0.20 wt%. Figure 6b illustrates the thermal conductivity ratio (TCR), *k_nf_/k_bf_* at varying temperatures. It is obvious that the TCR is increased by adding the MXene + Al_2_O_3_ nanocomposite. However, the maximum 61.8% enhancement in TC was observed at concentrations of 0.20 wt% and a temperature of 90 °C. Further addition of nanocomposites could increase the TCR, but, conversely, the stability of the nanofluids would deteriorate owing to the clustering effect.

#### Proposed Correlation for Thermal Conductivity

**Figure 7:**
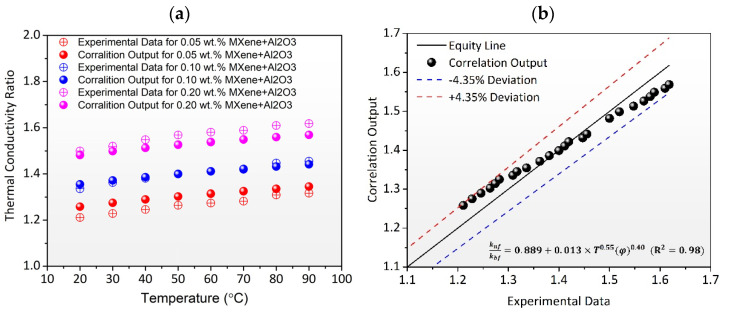
(**a**) Comparison between experimental TCR and correlation output as a function of temperature. (**b**) Correlation output vs. experimental TCR value with 4.35% deviation line.

The thermal conductivity of the TH55/MXene + Al_2_O_3_ nanofluid as a function of temperature and concentration is predicted using empirical correlation. Thermal conductivity correlation is developed using multivariate regression analysis using the thermal conductivity ratio as the dependent variable and temperature and concentrations as independent variables. In the temperature range of 20–90 °C, Equation (1) represents the proposed empirical correlation with R^2^ = 0.98. As illustrated in Figure 7a, the correlation output shows an excellent fit to the experimental data for the temperature range and concentration range studied. As shown in Figure 7b, the divergence between experimental and correlation output data is less than 4.35%, showing that correlations were quite accurate in predicting the TC of the nanofluid. The present correlation can be used to solve the TH55/MXene + Al_2_O_3_-assisted thermodynamic system with temperatures ranging from 20 to 90 °C. However, due to measuring instrument constraints, we were unable to quantify thermal conductivity at temperatures above 90 °C. However, it is anticipated that the proposed correlations would offer a satisfactory outcome when the temperature exceeds 90 °C.

(1) $$\frac{k_{nf}}{k_{bf}}=0.889+0.013\times T^{0.55}{\left(\varphi \right)}^{0.40}\left(R^{2}=0.98\right)$$

### 3.5. Dynamic Viscosity (µ) 

**Figure 8:**
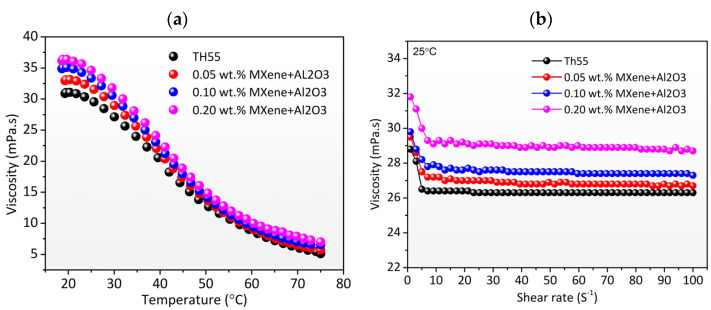
Dynamic viscosity as a function of: (**a**) temperature and, (**b**) shear rate or TH55 base fluid and TH55/ MXene + Al_2_O_3_ nanofluid at different concentrations.

The experimental *µ* vs. temperature and experimental *µ* vs. shear rate curves for TH55 and TH55+MXene + Al_2_O_3_ at various nanocomposite concentrations are shown in Figure 8a,b, respectively. As illustrated in Figure 8a, a shift in the viscosity curve is observed when the nanocomposite is added, and the temperature is varied. The shift indicates that the *µ* of the Therminol55 oil increased slightly with the inclusion of solid particles; however, substantially dropped at elevated temperatures for all the concentrations. The maximum increase in the *µ* is 17.14% for the concentration of 0.2 wt%, while it declined from 36.817 to 7.2 mPa as the temperature increased from 19 to 75 °C. As the temperature rises above 50 °C, the discrepancy between the pure Therminol55 and the nanofluids becomes almost insignificant. This is due to less flow resistance at higher temperatures between the nanoparticle and base fluid. Viscosity is a crucial property as it is directly associated with pumping power, pressure drop, and flow behavior of fluids in thermal systems [39]. For a dynamic thermal system, the low dynamic viscosity of the working fluid is preferred because it requires less pumping power and lowers running costs. Furthermore, the nanofluids exhibited Newtonian flow characteristics, as they stayed constant when the shear rate increased from 10 to 100 S^−1^ (see Figure 8b). In comparison to the rate of shear force, it also implies that TH55/ MXene + Al_2_O_3_ nanofluids are viscosity independent. The behavior of the nanofluids can be ascribed to the orientation of the fluid molecules to the rotation of the spindle [40]. Ref. [41] reported similar findings analyzing thermal oil/MWCNT–MgO nanofluids.

#### Proposed Correlation for Dynamic Viscosity

**Figure 9:**
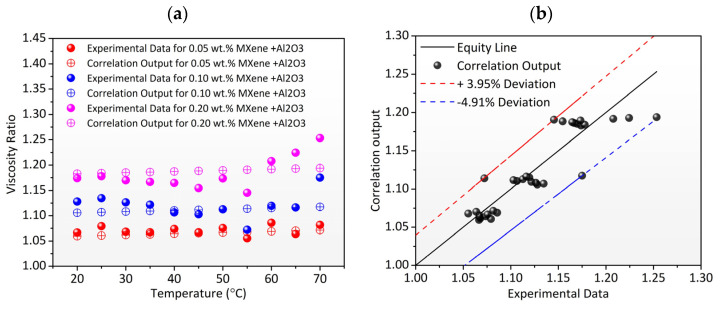
(**a**) Comparison between experimental viscosity ratio and correlation output as a function of temperature. (**b**) Correlation output vs. experimental viscosity ratio value with −4.91 and 3.95% deviation lines.

Using the same approach as indicated for thermal conductivity, empirical correlation is established to predict the characteristics of the TH55/MXene + Al_2_O_3_ nanofluid as a function of temperature and concentration. Empirical correlation is developed to predict the properties of the TH55/MXene + Al_2_O_3_ nanofluid as a function of temperature and concentration. Over a temperature range of 20–70 °C, Equation (2) provides the predicted empirical correlation with R^2^ = 0.95. With the exception of a few inconsistencies at higher temperatures, as shown in Figure 9a, the correlation output indicates an excellent fit to the experimental data over the temperature and concentration ranges tested. The discrepancy between experimental and correlation output data is (−) 4.91–3.95%, as shown in Figure 9b, indicating that correlations were highly accurate in predicting the nanofluid.

(2) $$\frac{\mu_{nf}}{\mu_{bf}}=1.279\times \varphi +\frac{e^{\left(2.37\times 10^{-4}\times T\right)}}{e^{\varphi^{1.56}}}\;\;\left(R^{2}=0.952\right)$$

### 3.6. Density

**Figure 10:**
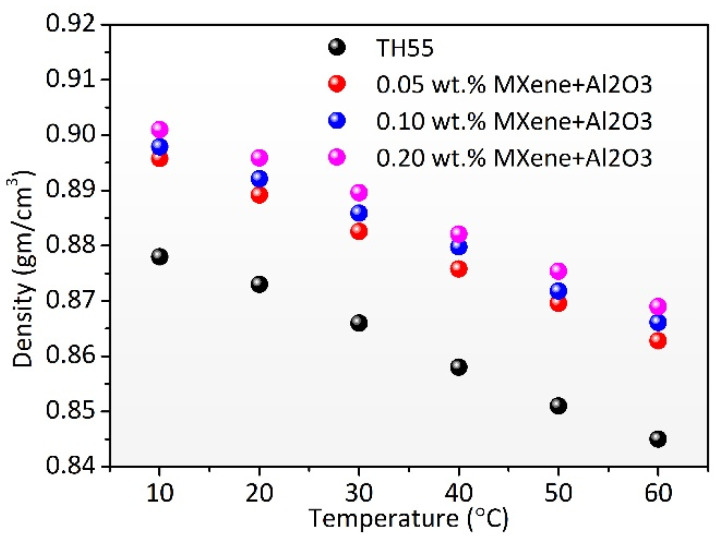
Density of TH-55/MXene + Al_2_O_3_ nanofluids with respect to varying temperature and particle concentrations.

The effective density of the working fluid influences convective heat transfer behavior and the outcome of the entire thermal system. TH-55/MXene + Al_2_O_3_ nanofluids showed an insignificant rise in density when compared with pure TH-55. Nevertheless, it decreased linearly at increasing temperatures (see Figure 10). The intermolecular attraction force is less at elevated temperature, which causes low density and viscosity of fluids, and similar research agrees [30]. Since the formulated nanofluid showed prominent viscosity and density at higher temperatures, it is expected to achieve considerable performance improvement using this nanofluid in thermal systems.

## 4. Concluding Remarks

The present study deals with the formulation of a new nanocomposite (MXene + Al_2_O_3_)-based nanofluid with Therminol55 as the base fluid at three different concentrations of 0.05, 0.10, and 0.20 wt%. The thermophysical, optical, and rheological properties were assessed and compared with those of the base fluid. Finally, new correlations were developed to predict the thermal conductivity and viscosity of the nanofluids. The formulated nanofluids had a satisfactory zeta potential value greater than −40 mV, confirming their better dispersion stability. FTIR analysis further confirmed the chemical stability, as no major chemical reaction was observed after introducing nanocomposite, save for mild oxidation. The thermal conductivity of the formulated nanofluid showed a maximum enhancement of 61.8% at 0.2 wt% and 90 °C together with an increase in viscosity of 17.14% at 20 °C, which substantially drops as the temperature increases. Furthermore, the thermal stability was not affected by adding nanocomposites as no significant discrepancy was observed between the TGA curves of base fluid and nanofluid. Two new correlations were proposed to predict the thermal conductivity and dynamic viscosity, respectively, which showed a good agreement with the experimental data.

## Figures and Tables

**Table 1:** Specifications of the MAX powder and Al_2_O_3_ nanoparticles.

Product	Purity	Size	Nature
MAX Powder	≥90%	≤40 μm	Hydrophilic
Al_2_O_3_ Nanoparticles	≥99%	~20 nm	Hydrophilic

## Data Availability

The data used for the analysis in this study are available within the article, while the datasets used or analyzed during the current study are available from the corresponding author upon reasonable request.

## Supplementary Materials

The following supporting information can be downloaded at: https://www.mdpi.com/article/10.3390/nano12111862/s1, Figure S1: Absorbance and transmittance spectra of TH55/MXene+ Al_2_O_3_ nanofluids as a function of wavelength from day 1 to day 14 for 0.05 wt% of MXene + Al_2_O_3_; Figure S2: Absorbance and transmittance spectra of TH55/MXene+ Al_2_O_3_ nanofluids as a function of wavelength from day 1 to day 14 for 0.10 wt% of MXene+ Al_2_O_3_; Figure S3: Absorbance and transmittance spectra of TH55/MXene+ Al_2_O_3_ nanofluids as a function of wavelength from day 1 to day 14 for 0.20 wt% of MXene + Al_2_O_3_.
