# Supplementary material for: Thermo-Optical Characterization of Therminol55 Based MXene–Al2O3 Hybridized Nanofluid and New Correlations for Thermal Properties
Source: Nanomaterials (Basel). 2022 May 30;12(11):1862. doi: 10.3390/nano12111862 (PMC9182443; doi:10.3390/nano12111862)
Supplement: Supplementary file 1 [file nanomaterials-12-01862-s001.zip › nanomaterials-1646108-supplementary.pdf]

Supplementary Materials

Thermo-Optical Characterization of Therminol55 Based MXene–Al_2O_3 Hybridized Nanofluid and New Correlations for Thermal Properties

Likhan Das [1], Khairul Habib [1,*], Kashif Irshad [2,3,*], Rahman Saidur [4], Salem Algarni [5] and Talal Alqahtani [5]

[1] Department of Mechanical Engineering, Universiti Teknologi PETRONAS32610 Seri Iskandar, Perak Darul Ridzuan, Malaysia; likhan.das11@gmail.com
[2] Interdisciplinary Research Center for Renewable Energy and Power Systems (IRC-REPS), King Fahd University of Petroleum & Minerals, Dhahran 31261, Saudi Arabia
[3] Researcher at K.A.CARE Energy Research & Innovation Center at Dhahran, Dhahran 31261, Saudi Arabia
[4] Research Centre for Nanomaterials and Energy Technology (RCNMET), School of Engineering and Technology, Sunway University, Petaling Jaya 47500, Malaysia; saidur@sunway.edu.my
[5] Department of Mechanical Engineering, King Khalid University, P.O. Box 9004, Abha 61413, Saudi Arabia; saalgarni@kku.edu.sa (S.A.); talqahtani@kku.edu.sa (T.A.)
* Correspondence: khairul.habib@utp.edu.my (K.H.); kashif.irshad@kfupm.edu.sa (K.I.); Tel.: +966-547854711(K.I.)

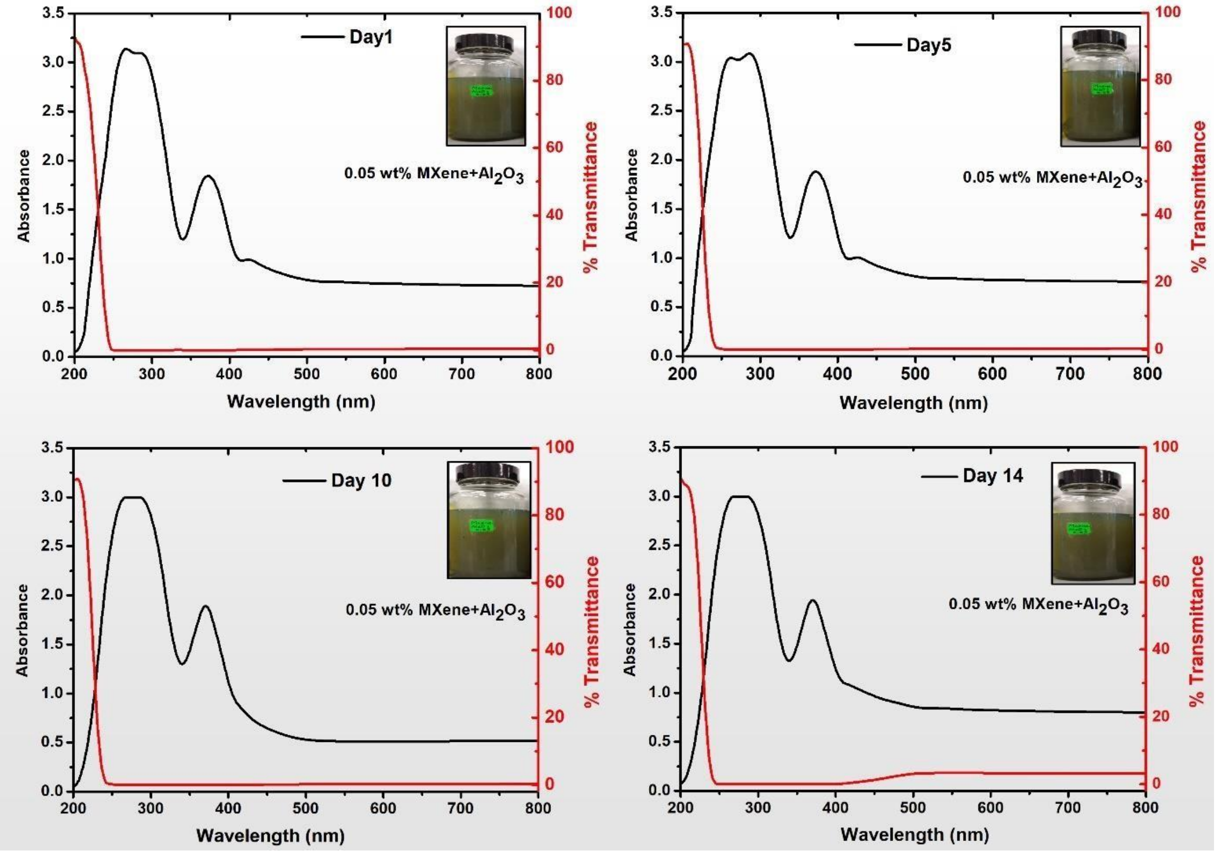

Figure S1. Absorbance and transmittance spectra of TH55/MXene+Al_2O_3 nanofluids as a function of wavelength from day 1 to day 14 for 0.05 wt% of MXene+Al_2O_3.

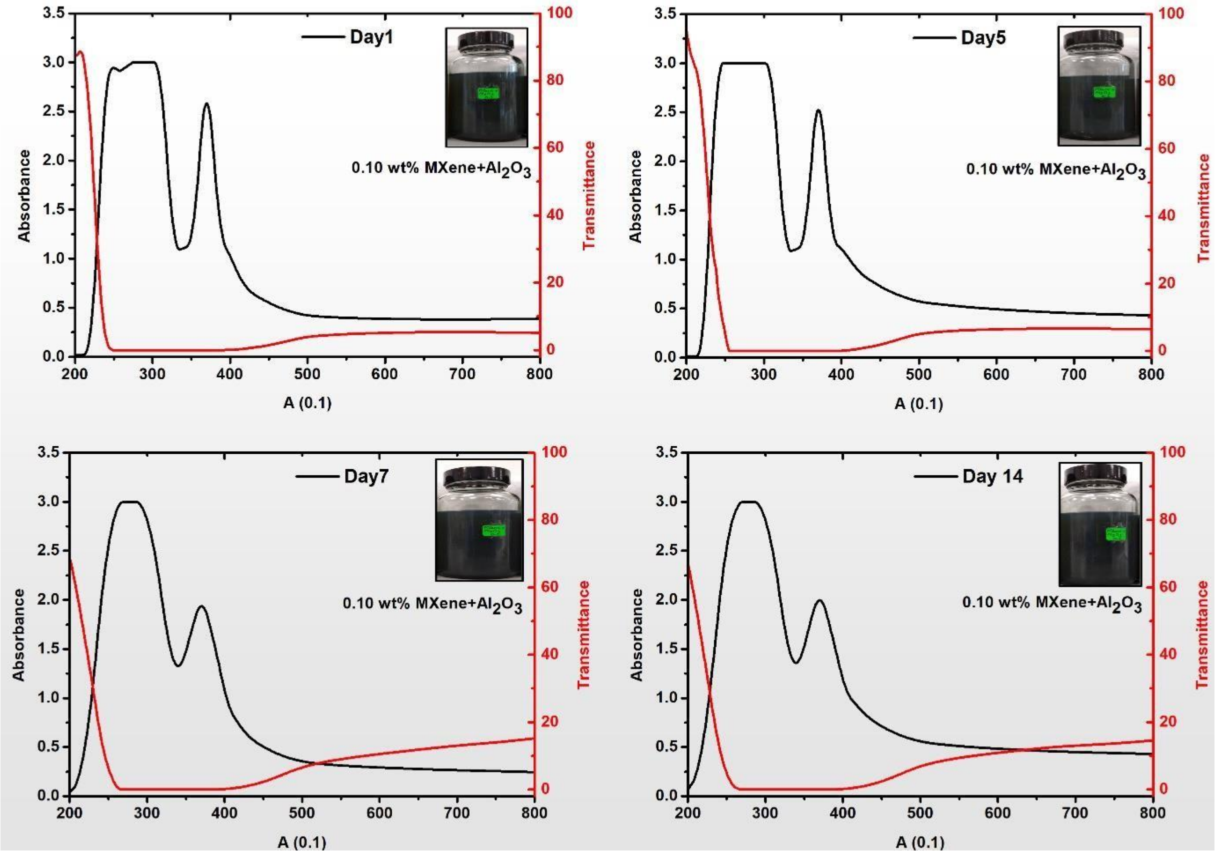

Figure S2. Absorbance and transmittance spectra of TH55/MXene+Al_2O_3 nanofluids as a function of wavelength from day 1 to day 14 for 0.10 wt% of MXene+Al_2O_3.

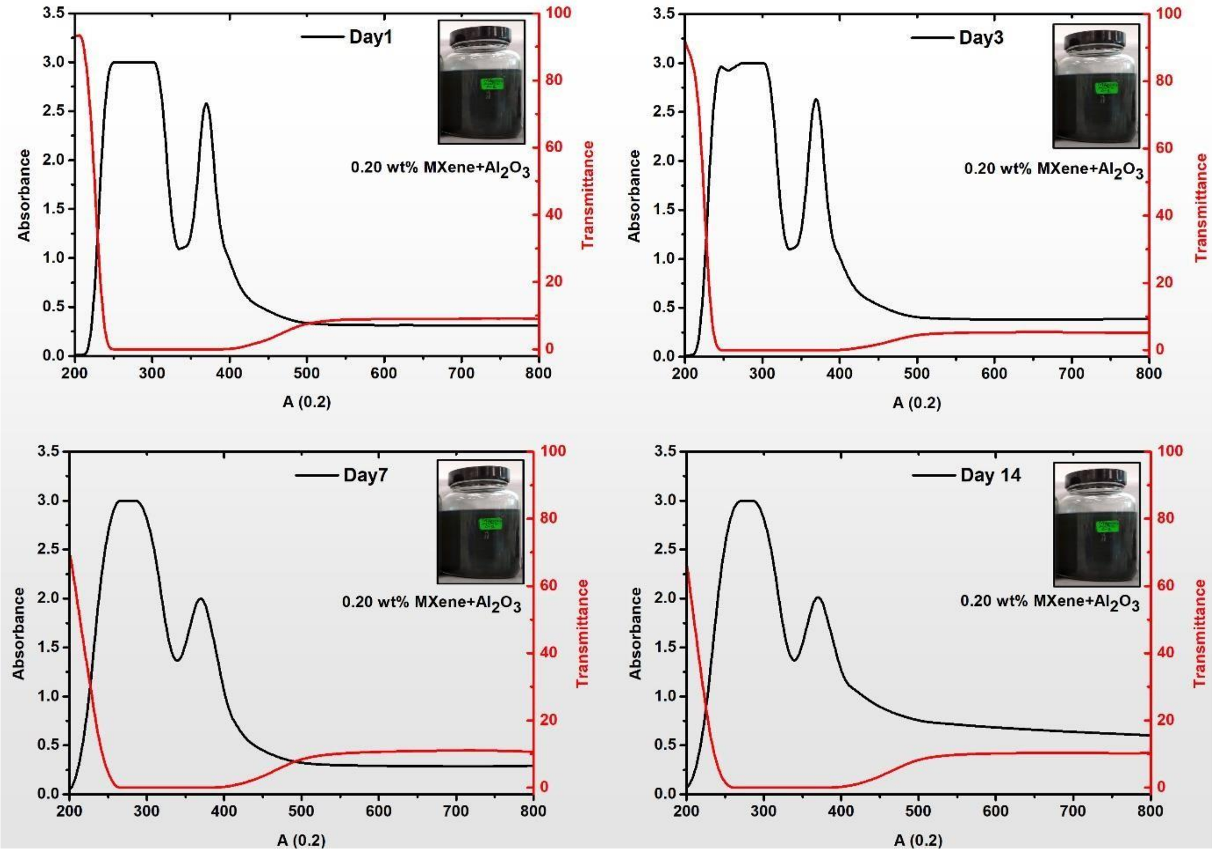

Figure S3. Absorbance and transmittance spectra of TH55/MXene+Al_2O_3 nanofluids as a function of wavelength from day 1 to day 14 for 0.20 wt% of MXene+Al_2O_3.
